# Bristol Medico-Chirurgical Society

**Published:** 1895-12

**Authors:** 


### Bristol Medico-Chirurgical Society.

*Annual Meeting, October 9th,* 1895.

Dr. A. J. Harrison, President, in the Chair.

After a vote of thanks to the retiring President (Dr. A. J. Harrison) had been unanimously passed, Mr. Arthur W. Prichard (the President-elect) took the Chair, and read his inaugural address (see page 241), for which a cordial vote of thanks was given him. The Honorary Secretary (Mr. J. Paul Bush) and the Assistant-Editor and Honorary Librarian (Mr. L. M. Griffiths) read their annual reports, which were adopted. The following officers were chosen for the ensuing year:—President-elect; Dr. A. E. Aust Lawrence: Honorary Secretary; Mr. J. Paul Bush: Honorary Librarian; Mr. L. M. Griffiths: Members of Committee; Dr. J. Michell Clarke, Mr. J. Dacre, Mr. W. H. Harsant, Dr. Bertram M. H. Rogers, Dr. J. E. Shaw, and Mr. G. Munro Smith: Representatives of the Society on the Committee of the Bristol Medical Library; Mr. L. M. Griffiths, Dr. G. Parker, and Dr. J. Swain.

---

*November 13th,* 1895.

Mr. Arthur W. Prichard, President, in the Chair.

Mr. C. A. Morton showed: (1) A man, aged 35, with cyst of the antrum, which had not caused general expansion of the bone, but presented as a soft swelling between the cheek and the superior maxilla. The contents had been withdrawn (a viscid yellow fluid, full of cholesterine crystals), and the opening in the outer wall of the antrum through which it had projected could be plainly felt; (2) A woman, aged 60, with a slowly-growing form of sarcoma of the superior maxilla, under treatment by injections of erysipelas toxins for four months. There had been a unilateral discharge of a minute quantity of pus from the nostril of the affected side many months before she came under observation, and the antrum had been explored to exclude empyema. A mass of new tissue an inch in thickness was then found, and the bony wall of the antrum had been absorbed so that it cut easily with the scalpel. The growth extended too far back to allow of removal. The result of treatment had been a diminution in the growth in the canine fossa, but it had spread over the nasal process. The pain had been much diminished. Mr. Morton described the origin and method employed in the erysipelas toxin treatment of malignant growths and gave Coley's results, and also referred to one other case in which he had employed the method.

—Mr. Charles A. Hayman thought that, as the fangs of the second (or thirteen year) upper molar teeth pierce the floor of the antrum in so many cases, the cyst under discussion may have been relieved permanently by extraction of the carious stumps now to be seen in the patient's mouth.—Dr. G. Parker said that he had used this toxin in three cases, and had learned a little about its mode of action. There are three methods of employing injections for malignant growths at present under discussion. Emmerich and Scholl use the filtered serum of animals which have been repeatedly injected with cultures of erysipelas; but their results are bitterly disputed in Germany, and Coley, who has recently tried the plan, has given it up. The same uncertainty exists as to the French method. Here injections are made of serum from animals repeatedly inoculated with the juice of malignant growths, and some success is claimed. Coley has gradually elaborated his own procedure: commencing in 1891 with living cultures of erysipelas as injections, then using the filtered products of the streptococcus mixed with a little of those of bacillus prodigiosus; while of late he utilises the bodies of the microbes also, and only sterilises the liquid at the moderate temperature of 58° C., which is not so high as to break up some of the delicate toxins contained in it. Moreover, he finds the greatest difficulty in growing a trustworthy toxin. His present ones are derived from a fatal case of erysipelas, and intensified by passing through rabbits. He has now treated eighty-four cases, but only forty-three of these were sarcoma, for which alone he recommends the treatment. Of these forty-three—many of them recurrent or tested microscopically—he claims that eleven are cured, besides three successes in carcinoma and two or three in epithelioma. Some half-a-dozen other cases are reported by his friends where the tumours have broken down and disappeared. Senn reports that he found it a complete failure when tried on nine cases; but it appears that only three were simple sarcomata, and besides he used toxins from various sources of doubtful quality. Coley is careful to inject the fluid near or into the growth; but when this is highly vascular, Dr. Parker finds that the greatest care is needed, otherwise even minute doses give a most dangerous reaction. In one of his cases, a patient of Mr. Barclay's, they had to do with a recurrent sarcoma, and here the remedy failed altogether, though a reaction of 101° and 103°, with rigors, headache, and vomiting, followed regularly. The second case was an internal tumour of doubtful character, and was remarkable for the absence of reaction, though as much as twenty minims were injected at once. In the third case—a recurrent carcinoma of the breast, widely disseminated—the toxins appeared to hold the growth in check for a time, and relieved the breathing; but the state of the patient was too feeble to permit the continuance of the treatment and the severe reactions. Dr. Parker realised that only one of his cases was a favourable one for trying the remedy; and in reviewing the large amount of evidence brought forward by Dr. Coley, the repeated microscopical examinations made, and the permanent recoveries met with in so many instances, he thinks that further trials may well be made in cases where operations are undesirable, however hopeless we may feel about such remedies in general.—Mr. W. R. Ackland thought in his experience that the anterior swelling of the face—which Mr. Morton thought was unusual—indicated that the cyst of antrum was only secondary to cyst of tooth. He would like further to say, as to treatment, that most of these cases fill up again. Packing with sea-tangle or carbolised gauze was usually necessary.—Mr. Greig Smith said that the treatment of tumours by toxins rested on a *vera causa*—the undoubted fact that tumours occasionally disappeared after an attack of erysipelas. He was of opinion, however, that the treatment had not yet shown itself worthy of support. The results at the best were poor: some of them rested on doubtful diagnosis, and a few on more than doubtful diagnosis. Now, every such case should be based on a unanimous diagnosis by a number of surgeons. It seemed a pity to waste a single case of experiment in this treatment: there was a superabundance of undoubted material, and he urged that only cases of undoubted diagnosis should so be treated. He personally doubted if Mr. Morton's case was sarcoma at all—as did others. He would wait for more definite and more favourable results before employing it in his own practice.—Dr. Markham Skerritt inquired in what way the erysipelas toxin was supposed to influence new growths—whether by a direct specific effect upon their nutrition, or indirectly as a result of the fever set up. Such an apparent influence was not limited to tumours. Cases were on record in which the course of pulmonary phthisis had been arrested after an attack of erysipelas. Nor was it exercised by erysipelas alone. It was possible that the type of an existing disease might be altered after any intercurrent acute febrile attack. He instanced a case of phthisis which had recently come under his own observation, in which the symptoms and physical signs all underwent most striking improvement after an attack of influenza with high temperature, the tubercular process seeming to lose all its activity. Such evidence went to support the view that any influence of erysipelas upon tumours was due to the accompanying pyrexia rather than to the specific power of its toxins. It would probably follow that there would be no certainty of result where a tumour was attacked by erysipelas or treated with the toxin of this disease; and perhaps the short-lived fever accompanying the artificially-produced reactions would be less likely to exert an influence than the prolonged and high pyrexia of a severe attack of ordinary erysipelas.—Mr. Munro Smith remarked on the great difficulty of making a certain diagnosis in cases of this nature, and narrated a case which had been operated on eighteen months ago by Dr. James Swain for tumour of the neck which showed all the microscopic appearances of small round-celled sarcoma. This recurred, and Mr. Munro Smith attempted to remove it; but the operation had to be abandoned, owing to the depth and adhesions of the tumour, which spread down to the front of the vertebræ. Slight suppuration with temperature of 101° followed; and since the operation, four months ago, the large tumour has entirely disappeared.—Mr. W. M. Barclay recalled the case of a man in the Bristol General Hospital in which a large tumour in the buttock was diagnosed as a sarcoma, and an operation accordingly undertaken. The growth extended too far for extirpation, and the attempt was given up. The growth, microscopically, was a round-celled sarcoma. It nevertheless entirely disappeared, the wound healing by first intention. (These remarks were made in reference to the uncertainty of diagnosis in malignant cases, even when this seems complete in every point.)—Dr. Watson Williams drew attention to the value of trans-illumination by means of an electric light in the mouth, as an aid to diagnosis in certain diseases of the antrum. Referring to the case treated by erysipelas toxin, he mentioned a case of inoperable epithelioma of the throat which he had attempted to treat by inoculating erysipelas streptococci two years ago. He suggested that possibly one might get better results by frequent and repeated small injections, instead of the larger injections which aimed at the production of a definite febrile reaction. In 1891 he had advocated this method of giving tuberculin in tubercular disease; and a limited experience tended to show that by *avoiding* the "reaction" one might get better results than by the more usual method.—Dr. A. J. HARRISON, before accepting the beneficent effects of erysipelas—be it streptococcus or its toxin—would like to point out that many cases of lupus have been cured or greatly improved by an attack of erysipelas, and that probably the benefit arises from the febrile condition that is induced.—The PRESIDENT referred to a case under the care of Dr. James Swain, who was unable to be present at the meeting. A man, aged 48, suffered from inoperable recurrent sarcoma (diagnosis confirmed by microscope) of the right upper jaw. He was injected almost daily for nearly five weeks with the sterilised products of the streptococcus erysipelatis and the bacillus prodigiosus, after Coley's method. Injections under the healthy skin caused some headache and a feeling of *malaise*, but into the sarcomatous tissue were generally followed by a distinct reaction, with an elevation of temperature (100° to 103° F.), and followed in a day or two by necrobiosis of the tumour in the immediate neighbourhood of the injection. Rigors were fairly frequent, and much depression ensued. In spite of the "breaking down" which occurred, the growth gradually increased, and the patient determined to give up the treatment. He died in September last—about three months after the injections were discontinued.[1] The case was no doubt one of the least favourable for this method of treatment; but though unsuccessful, it seemed to give some promise for the future. Certainly in such deplorably hopeless cases as those of inoperable malignant disease, we should give a fair trial to this new form of therapeusis; but in order not to arrive at false conclusions, it is necessary that the diagnosis should in all cases be supported by microscopical examination.—In reply, Mr. MORTON considered that the origin of such a cyst in disease of the root of the second molar was quite unlikely. These cysts were true cysts, not dropsy of the antrum as used to be taught. A diseased second molar root penetrating the antral wall would be much more likely to cause empyema. The cyst, he thought, probably arose in the antral wall and had no connection with the teeth: such cysts might be seen hanging like clusters of grapes from the wall in some specimens. Mr. Morton agreed with Dr. Parker in the uncertain and varying results of the erysipelas serum as compared with Coley's toxins; and stated that in the preparation he had used, the streptococci had been killed by heating to 58° C., and the toxic products were mixed with those of bacillus prodigiosus. He could not accept the view that the disappearance of malignant growths during an attack of erysipelas was a mere coincidence, and quoted the statistics of such cases in support of the belief; nor could he admit that the swelling was inflammatory rather than new growth, as the amount of new tissue was so great, and the bony wall of the antrum had been absorbed by it. Moreover, there was nothing to account for any inflammatory condition. The view put forward by Dr. Skerritt that possibly the high temperature of erysipelas was the active agent in the destruction of the growth, he could not accept, or why did not growth disappear during other conditions of continued high fever? In reply to Dr. Williams, he said he should not care to employ the toxin in malignant growth of the larynx, injected into the submucous or cellular tissue in the neighbourhood, without being prepared for tracheotomy, as the œdema produced was often very great.

Dr. MICHELL CLARKE showed a man, aged 54, who had suffered from Huntington's chorea for five years. The onset was gradual.

1 Further details of this case have since been published. See *Brit. M. J.*, Dec. 7.

His father was choreic and insane, and his brother is choreic and in an asylum. Other near relatives were choreic and insane. The spasms affected nearly all the muscles of the body, but ceased during sleep. The organic functions were not interfered with. Dr. Clarke mentioned another case of the same affection formerly under his care. The patient was about 50, and the spasms were sudden and shocklike. Others of the patient's relations were affected. Some account was given of the recent researches into the morbid anatomy of the disease.

Dr. F. H. Edgeworth showed a series of lantern slides, prepared by Mr. James Taylor, illustrating the pathological changes which occur in the thyroid in Graves's disease. To emphasise the characteristics of these changes, photographs of the normal gland and of adenoma of the thyroid were also demonstrated.

J. Paul Bush, *Hon. Sec.*

